# Three-Dimensional (3D) Printed Microneedles for Microencapsulated Cell Extrusion

**DOI:** 10.3390/bioengineering5030059

**Published:** 2018-07-31

**Authors:** Chantell Farias, Roman Lyman, Cecilia Hemingway, Huong Chau, Anne Mahacek, Evangelia Bouzos, Maryam Mobed-Miremadi

**Affiliations:** 1 Department of Bioengineering, Santa Clara University, Santa Clara, CA 95053-0583, USA; cfarias@scu.edu (C.F.); rlyman@scu.edu (R.L.); chemingway@scu.edu (C.H.); hchau@scu.edu (H.C.); ebouzos@scu.edu (E.B.); 2 SCU Maker Lab, Santa Clara University, Santa Clara, CA 95053-0583, USA; amahacek@scu.edu

**Keywords:** hollow microneedles, 3D printing, stereolithography, alginate, microencapsulation, cell delivery, HepG2 cells, biofabrication, wound healing, sustainability

## Abstract

Cell-hydrogel based therapies offer great promise for wound healing. The specific aim of this study was to assess the viability of human hepatocellular carcinoma (HepG2) cells immobilized in atomized alginate capsules (3.5% (*w*/*v*) alginate, d = 225 µm ± 24.5 µm) post-extrusion through a three-dimensional (3D) printed methacrylate-based custom hollow microneedle assembly (circular array of 13 conical frusta) fabricated using stereolithography. With a jetting reliability of 80%, the solvent-sterilized device with a root mean square roughness of 158 nm at the extrusion nozzle tip (d = 325 μm) was operated at a flowrate of 12 mL/min. There was no significant difference between the viability of the sheared and control samples for extrusion times of 2 h (*p =* 0.14, *α* = 0.05) and 24 h (*p =* 0.5, *α* = 0.05) post-atomization. Factoring the increase in extrusion yield from 21.2% to 56.4% attributed to hydrogel bioerosion quantifiable by a loss in resilience from 5470 (J/m^3^) to 3250 (J/m^3^), there was no significant difference in percentage relative payload (*p =* 0.2628, *α* = 0.05) when extrusion occurred 24 h (12.2 ± 4.9%) when compared to 2 h (9.9 ± 2.8%) post-atomization. Results from this paper highlight the feasibility of encapsulated cell extrusion, specifically protection from shear, through a hollow microneedle assembly reported for the first time in literature.

## 1. Introduction

Due to the growing need for minimally invasive drug delivery systems and the concern of causing pain and anxiety in patients using a conventional hypodermic needle, localized, and generally pain-free delivery systems for therapeutics, such as resorbable microneedle (RMN) patches and hollow microneedle (HMN) arrays have been developed. Since the growth of the microelectronics industry in the 1990′s and the success of microneedle fabrication and transdermal drug administration in 1998, the development of micromolding techniques for dissolving polymeric microneedle fabrication has enhanced the payload of delivery systems, prompting researchers to expand the repertoire of microneedle therapeutic uses [1]. Transdermal drug delivery studies against complex diseases and vaccination include those of Wang, et al. [2] and Ye., Y. et al. [3], who have loaded hyaluronic acid (HA)-based microneedles with anti-PD1 antibody, glucose oxidase (GOx)-encapsulated dextran nanoparticles, and tumor lysate, melanin, respectively, for the transdermal delivery of antitumor immune response-promoting compounds against melanoma; as well as those of Hu, et al. [4], who loaded HA-based microneedles with insulin and GOx-encapsulated polymeric vesicles, and Lahiji, et al. [5], who developed dissolving microneedles encapsulated with Exendin-4 hormone, against types I and II diabetes. A more comprehensive list of diseases that are treated and therapeutic compounds delivered using MN technology is presented in Table S1 [2,3,4,5,6,7,8,9,10,11,12,13,14,15,16].

Across multiple physiological time scales associated with wound type and wound healing [17] the use of MNs has been investigated in the following areas: (a) antimicrobial wound treatment [18,19,20]; (b) proliferation of endothelial cells [21,22]; (c) scar repair [22,23]; and, (d) treatment of chronic wounds and burns [24,25]. Related cutting edge applications of MNs include biointerfacing [26], wound detection [27], and smart bandages under development combining detection and delivery [28].

Micromolding is the most widely manufacturing method used [12,29]. Other methods include direct photolithography [30], drawing lithography [31], solvent casting [32], mold-based etching [33], and lithography [34], which are all variants that are developed based on the micromolding method of polydimethylsiloxane (PMDS) polymer casting. Numerous biofabrication methods have been devised for RMNs and HMNs a subset of which will be elaborated upon below and presented in Table S2. Ruggiero, et al. [35] have adapted an electro-drawing technique of fabrication, by designing a pattern distribution of integrated metallic micro-heaters that applies an electrohydrodynamic (EHP) force onto polymer solution sessile drops for microneedle shaping. Their setup induces the generation of a uniform electric field, allowing for homogeneous EHP deformation of the drops and careful formation into tapered poly-lactic-glycolic acid (PLGA) RMNs. Johnson, et al. [36] have used an additive manufacturing (AM) method, Continuous Liquid Interface Production (CLIP), to three-dimensional (3D) print dissolving microneedle structures from a computer aided design (CAD) file. The CLIP method they have adapted photopolymerizes the MNs by selectively targeting and solidifying photoreactive resin with ultra-violet (UV) light on a rising platform [36]. In addition, Huh, et al. [37] adapted a Droplet-borne air blowing (DAB) method by first dispensing a polymer drug mixture as a droplet, then contacting, drawing, air-drying, and separating the mixture to form microneedle structures, and similarly, Kim, et al. [38] designed a “Dipping” method to form RMNs in which polymer-coated pillar tips are first coated in a drug of interest, dipped in drug-unmixed solution, then lifted, air-dried, and separated to form microneedle tips. Most recently, Luzuriaga, et al. [39] have adapted a fused deposition modeling (FDM™) based printing method to fabricate RMN structures; this AM method circumvents the need for a clean-room, master template, or mold, which are mandatory for previously used micromolding and lithography-based techniques for fabrication [40,41], and introduces sustainability into microneedle array manufacturing.

Although 3D printing for MNs was first investigated in 2007 by Ovsianikov et al. [42] while using a lithography-based multiphoton polymerization printing method, the ability to print biocompatible and biodegradable materials from conventional 3D printing methods, such as stereolithography (SLA), FDM™, Selective Laser Sintering (SLS), CLIP, and Digital Light Processing (DLP) [43,44,45] has been the focus of numerous studies presented in Table S3. The biofabrication methods have been classified under the AM processes outlined in ISO/ASTM 52900:2015 [46]. SLS and primarily SLA 3D printing have been of interest due to their high resolution and their ability to fabricate solid features smaller than 100 μm, which are ideal for microneedle parameters. However, their disadvantage is that untreated photopolymers used in commercial 3D printers have a lack of biocompatibility and can therefore be toxic for living cell compounds [47,48,49,50]. Therefore, material selection in SLA and SLS 3D printing of MN arrays is key as successfully reported in the following studies: Kang, et al. [51] and Lu, et al. [52] have successfully printed MN arrays pre-loaded with drugs using SLA 3D printing. While, the former study encompassed biofabrication of poly (ethylene glycol) diacrylate (PEGDA) based MN structures embedded in bovine serum albumin, the latter study was based on printing results of poly (propylene fumarate)/diethyl fumarate (PFF) MN arrays that are loaded with dacarbazine drug. In addition to polymers, Gieseke, et al. [53] have SLS-3D printed stainless steel 316l alloy. As previously mentioned, FDM™, followed by etching post-printing used for RMN fabrication [39], its main limitation being the lower resolution when compared to other printing methods, such as SLA and SLS in the absence of post-processing. Other studies, such as Johnson, et al.’s [36] and Miller, et al.’s [54], have also investigated MN fabrication using CLIP and DLP 3D printing methods, respectively, to print biocompatible acrylate-based photopolymer materials, such as Trimethylolpropane Triacrylate and eShell 200 photosensitive acrylate-based biocompatible polymer resin by EnvisionTEC, Inc. (Dearborn, MI, USA). 

Due to the breadth of applications, and design considerations for optimal delivery and minimal patient pain, numerous geometries, and array dimensions of MNs have been investigated a subset of which will be elaborated upon. A synopsis is presented in Table S4, with reference to geometries in MN studies presented in Tables S1–S3. MN structures range over the following spatial configurations: Pointed tip with triangular slope [54], conical shape [2,4,14,55,56], cylindrical shape [57], cylindrical body and pointed head [45,52,58], tip-beveled triangular cylinder shape [34], tip-beveled cylinder shape [59,60], pyramidal shape [8,12,61], and tetrahedron-shaped [62]. Based on the studies, these different microneedle shapes and geometries have been shown to breach the stratum corneum layer of human and porcine skin.

Gupta, et al. studied microneedle infusion pressure and pain in human patients while using saline and hollow glass microneedles of variable insertion depth [63]. According to their study, pain is minimized with maximal microneedle insertion depth of 750 μm, delivered medium saline volume of 1 mL, and infusion flow rate 0.3 mL/min. Furthermore, pressures of approximately 500, 1000, and 2000 mmHg were optimal for minimized pain for microneedle insertion depths of 500 and 700 μm. In addition, microneedle tip geometry was investigated in relation to pain; applying microneedles of up to about 700 μm in length, and up to 55 degrees pitch to the skin [64], and using either an array of 10 × 750 μm tall, 75 μm thick, 200 μm wide or 50 × 500 μm tall, 75 μm thick, 200 μm wide microneedles [65] were reported to generally cause minimal pain (no pain or light pain) in patients.

Captured across multiple studies are the Mesenchymal Stem Cells demonstrated effects on cutaneous wound healing and skin regeneration in vitro and in vivo models, by accelerating wound closure, enhancing re-epithelialization, increasing angiogenesis, promoting granulation tissue formation, modulating inflammation, and regulating extracellular matrix remodeling [66,67,68,69,70,71,72]. There are currently 142 registered human clinical trials on stem cells and wound healing worldwide [73]. The major obstacle to clinical translation is stem cell immunogenicity and their reduced survival in vivo [74,75,76,77]. To this effect, polymeric stem cell entrapment strategies, including scaffold surface modifications for transdermal delivery have been devised and tested in preclinical animal wound models [78]. Scaffolding materials include natural, synthetic, and composite polymers and hydrogels with tunable micro/macroporosities, stiffness and bioerosion rates [79,80,81] Amongst nanoporous immuno-protective biomimetic hydrogels, the encapsulation of cells in alginate a linear unbranched polysaccharide containing β-d-mannuronic acid (M) and α-l-guluronic acid (G) residues has been widely applied in preclinical models of drug delivery, wound healing, and tissue regeneration as early as the 1960s [82,83,84,85,86,87].

In a recent British human pilot study to test the safety and efficacy of microneedles for cell delivery ex vivo in patients with vitiligo, melanocyte, keratinocyte, and mixed epidermal cell suspensions were successfully extruded through various types of hollow silicon microneedles ranging from 75–100 μm in bore size and 400–700 μm in depth. All cell types investigated maintained their distinctive phenotype after extrusion through microneedles and at all concentrations after 72 h of testing [88,89].

Combining the advantages of MN-based delivery systems, 3D microenvironments for cell survival and the presence of an immuno-isolation barrier, a custom 3D-printed hollow MN device has been designed to extrude alginate microcapsules into which epithelial cells have been seeded. Human hepatocellular carcinoma (HepG2/ATCC HB-8065), which is a shear sensitive cell line, has been chosen to simulate the behavior of the cocktail of epithelial cells [90,91,92]. To date, there is no study capturing the preclinical biomaterials characterization of HMNs fabricated using stereolithography through which encapsulated mammalian cells have been sheared. Therefore, the specific aim of this paper is to determine whether there is a difference in viability between the extruded encapsulated cells as compared to non-extruded controls.

## 2. Materials and Methods

### 2.1. Materials

Human hepatocellular carcinoma (HepG2/ATCC HB-8065) and human glioblastoma (U-87 MG/ATCC HTB-14)™ were obtained from ATCC (Manassas, VA, USA). Dulbecco’s modified Eagle medium (DMEM, Life Technologies 11965) and fetal bovine serum (FBS, Life Technologies 16000) were procured from Life Technologies (Carlsbad, CA, USA). Penicillin-streptomycin (Cellgro, 30–002-CI) and Trypsin-EDTA (Cellgro 25–053-CI) were manufactured by Cellgro (Manassas, VA, USA). All of the reagents that are required for the microencapsulation of cells, including medium viscosity alginate (Sigma A2033 (*μ* > 2000 cP, Mv = 900–1000 kDa, M/G ratio 1.6), blue dextran dye (Sigma, D4772), as well as reagent grade salts and solvents were purchased from Sigma Aldrich (St. Louis, MO, USA). Proprietary 3D printing photoresins were provided by Formlabs (Summerville, MA, USA).

### 2.2. Methods

**Figure 1:**
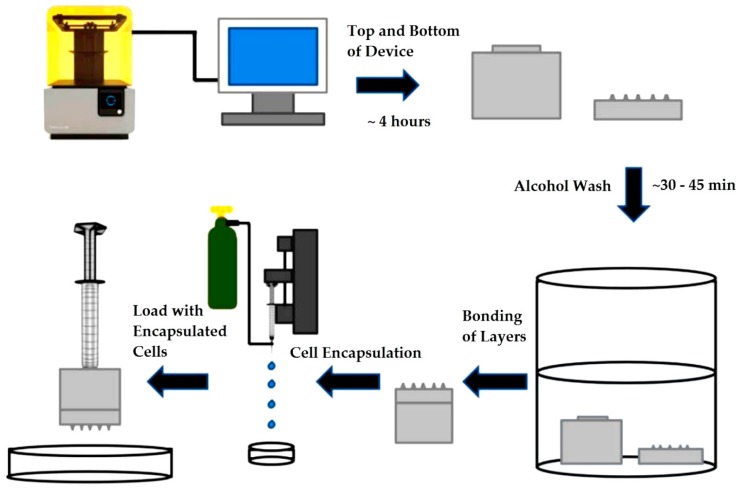
Experimental process flow steps of device fabrication, cell encapsulation, and microcapsule extrusion.

As shown in Figure 1, is the experimental process flow beginning and ending by the 3D printing step and microcapsule extrusion, respectively. SolidWorks was used to design the device that was 3D printed using a Formlabs 2 printer with subsequent post-processing steps. Bonding of the two layers was accomplished using a silicone-based sealant GE Silicone II* Caulk, (General electrics, Boston, MA, USA). The cells were encapsulated in sterilized (3.5% (*w*/*v*)) alginate solution. Following encapsulation and crosslinking with 1.5% (*w*/*v*) CaCl_2_, the microcapsules were extruded through the HMN biodevice using a 1 mL syringe.

#### 2.2.1. Device Design Printing and Assembly

Multiple screening runs were conducted prior to determining the nominal MN dimensions, in order to reach a compromise between reported minimization of patient pain [63,64,65], and the resolution limit of the 3D printer. The choice of MN material selection was dictated, in turn, by the highest theoretical resolution of 25 μm achievable using a single photoresin formulation. Hence, prototype replication, biomaterials characterization, extrusion, and viability testing was only conducted for the design discussed in the following section.

(1) Design

The design of the MN device structure was comprised of two main parts: one containing the array of HMNs and the other consisting of a fluid chamber (reservoir).

**Figure 2:**
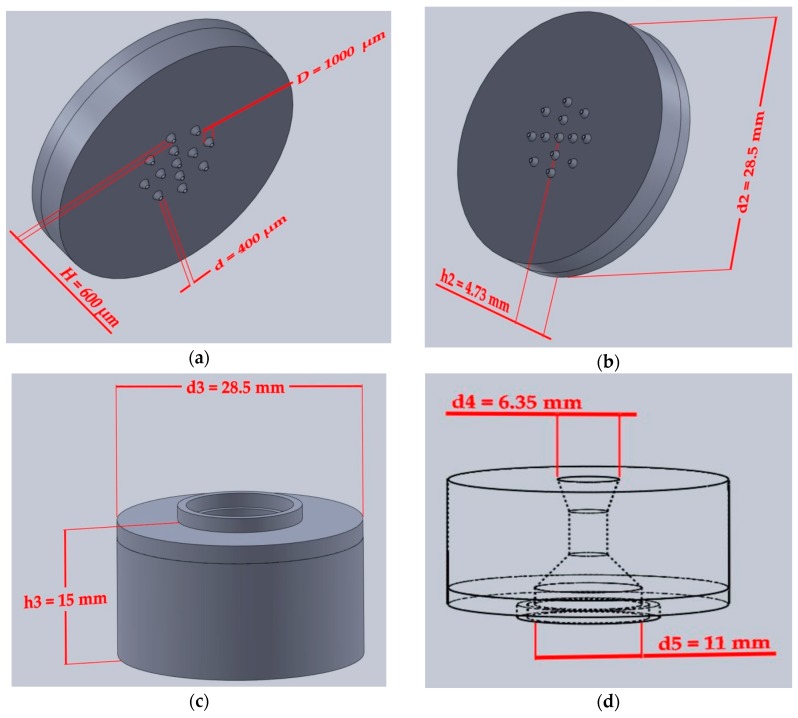
SolidWorks drawings of the printed device with corresponding dimensioning. (**a**) Circular array of 13 hollow microneedles; (**b**) bottom layer of device; (**c**) top layer of device consisting of a large cylindrical outer shape; and, (**d**) Side view of device rotated 180° with respect to Figure 2c illustrating the reservoir chamber.

The device prototype was designed using SolidWorks 2017 software (Dassault Systèmes, SolidWorks Corporation, Waltham, MA, USA). Formlabs Clear^®^ photoresin (FLGPCL02) [93] was used to create the top and bottom layers of the device. Reflected below are the nominal dimensions of the biodevice. The bottom layer (Figure 2a,b) included a 28.5 mm diameter (d2) flat disc of height 4.73 mm (h2) with a circular array of 13 microneedles. The dimensions of the cones are: D the diameter (D = 1000 μm); d, the tip diameter (d = 400 μm), H the height (H = 600 μm).

The top layer (Figure 2c) consisted of a large cylindrical outer shape of diameter 28.5 mm (d3) and height of 15 mm (h3). Shown in Figure 2d, is the inner reservoir chamber that is characterized by a volume of 0.5 mL, with a top opening diameter of d4 = 6.35 mm through which the syringe is inserted and bottom opening diameter of d5 = 11 mm.

(2) 3D Printing

3D printing was executed using Formlabs Form 2 printer (Formlabs Inc., 2018, Somerville, MA, USA), operating upon the principle of SLA, with a maximum resolution of 25 μm.

To set the orientation of the device and to generate supports, the preprocessing software, PreForm (Formlabs Inc., v2.16.0, Somerville, MA, USA) was used. This software confirmed the scale of the part, the orientation, the placement of the build tray, as well as the resolution for the photoresin to be used. PreForm enabled mobility of supports, once generated, to ensure that they were not placed at critical feature points of the device. Once these parameters were verified, the device was sent to the printer.

During the printing process, UV light was directed through the window on the bottom side of the printer and selectively cured each cross-section. The microneedle device that is discussed in this paper was orientated so that the microneedle orifices would be perpendicular to the support system. This orientation was possible due to their small dimensions, had the orifices been larger and more susceptible to collapse, they would have to be oriented at an angle to reduce the surface area of each cross-section. Once the parts were successfully printed, supports were removed using standard pliers and the parts were placed in an alcohol bath to ensure quality resolution.

Specifically, parts were placed in isopropyl alcohol (IPA, 70% *v*/*v*)), bathed for 20–30 min (10–15 min in tank 1, 10–15 min in tank 2), and then placed in a UV unit for 15 min for post-curing and removal of the uncured resin by leaching. The total processing time for 3D printing and washing the parts took over 5 h, with the ability to print three assemblies per batch.

A total of three devices to be assembled were printed and inspected for dimensional analysis, using a transmission microscope (Olympus CKX53, Center Valley, PA, USA) specifically for the tip radius, r. However, a single assembled part was used throughout experimentation.

(3) Sterilization

Prior to the assembly of the top and bottom parts of the device, the parts were soaked in ethanol (70% *v*/*v*) followed by three rinses in sterile DI water. This process will be referred to throughout the manuscript as solvent sterilization.

(4) Assembly

The top and bottom part of the device were mounted in a sterile environment using a silicone-based sealant GE Silicone II* Caulk, (General electrics, Boston, MA, USA). The parts to be sealed were maintained at 50 psi for 30 min.

#### 2.2.2. Surface Topography

Prior to imaging, the samples were pre-cleaned using an air pump (DOA-P704-AA, Gast, MI, USA) adjusted to 50 psi in order to reduce the risk of particulate contamination in the cleanroom environment. Imaging was performed via a 3100 Dimension atomic force microscopy machine (DAFM-XYZ, Bruker Instruments, Billerica, MA, USA). The Atomic Force Microscopy (AFM) scan was conducted in tapping mode using a Pyrex-Nitride probe (PNP-TR-20, NanoWorld, Neuchâtel, Switzerland) with triangular cantilever (resonant frequency 17 kHz, force constant 0.08 N/m), thickness 500 nm, length 200 μm, tip radius 7–10 nm). Nanoscope v6.13 (Bruker Instruments, Billerica, MA, USA) and Gwyddion v2.3 (Czech Metrology Institute, Brno, Czechoslovakia) were used as qualitative real-time and quantitative image analysis software, respectively. Section analysis and roughness analysis were conducted on the regions of the device that were designed to contact (squares 1 and 2 in Figure 4a) and penetrate the skin (Figure 4c). The scan sizes were 6 μm and 2.8 μm for the flat part and the tip of the microneedle, respectively. Scans were conducted at a frequency of 0.886Hz. 

A custom piece comprised of a rectangular array of 18 MNs (Figure 4a) instead of a circular array of 13 MNs (Figure 2a) with identical frusta dimensions being printed for relative ease and accuracy of XY stage positioning when indexing from feature to feature.

It was assumed that the surface topography of this custom part is representative of that of the biodevice.

#### 2.2.3. Mass Flow Capability and Leakage Test 

Prior to wet testing conducted at 20 °C, parts were inspected for nozzle occlusion and particulate residue using a transmission microscope (Olympus CKX53, Center Valley, PA, USA). When necessary, nozzle obstruction was alleviated using an air pump (DOA-P704-AA, Gast, MI, USA) adjusted to 50 psi. Flow testing was conducted according to a modified procedure for HMN administration to patients [63].

A micropipette (3124000121, Eppendorf, Hamburg, Germany) was used to transfer 0.5 mL of sample into the MN reservoir. Flow testing was conducted at two average flowrates of 1.2 mL/min and 12 mL/min. Blue dextran (single phase) and microcapsules (two phase capsules/media) were used to grade the size of the opening and capture the open/blocked states of the MNs. For the lower flowrate (1.2 mL/min), a syringe pump (NE-1000, New Era Systems, Farmingdale, NY, USA) housing an empty sterile and graduated 1 mL syringe filled with air (Becton Dickinson, Franklin Lakes, NJ, USA) connected to the top of reservoir was used to push the sample through. At the higher flowrate (12 mL/min), the air filled 1 mL syringe was pushed manually and the injection was recorded while using a vision system. Specifically, image capture was conducted at 1 fps using a color camera (1.3 MPX) with a 12.5 mm lens (MA 732, Biomomentum Inc., Montreal, QC, Canada). The flowrate was determined by counting the images until the air-filled syringe was empty.

Percentage jetting reliability was defined as the average number of open nozzles through which two-phase fluid was extruded over 10 runs.

#### 2.2.4. Cell Culture

Two epithelial cell lines (HepG2 and U87-G cells) were maintained following standard mammalian cell culture practices (ATCC, Manassas, VA, USA). The cells were cultured in Dulbecco’s modified Eagle medium (DMEM) (Mediatech, Manassas, VA, USA) and supplemented with 10% fetal bovine serum (FBS) Life Technologies, Carlsbad, CA, USA), sodium pyruvate (Life Technologies), MEM non-essential amino acids (Life Technologies), and 1% penicillin-streptomycin (CellGro, Manassas, VA, USA). They were incubated at 37 °C in a 5% CO_2_ humidified environment and then grown in 100 mm tissue culture dishes (Greiner, Bio-One, Monroe, CA, USA) to 60–80% confluency. They were subcultured at a 1:4 ratio with 0.25% trypsin (CellGro, Manassas, VA, USA).

#### 2.2.5. Cytotoxicity Screening

Cytotoxicity screening was conducted according to ISO 10993-5 guidelines [94]. Following solvent sterilization, a 5 mm thick strip of 1 × 3 mm of custom 3D printed part was submerged in cell media containing 3 × 10^4^ cells/mL passaged U87-G cells with a tissue culture polystyrene dish (TCPS) that was prepared with the same cell density as control. Confluency was examined at 24 h using microscopic inspection.

The higher surface area to volume ratio (S/V) of the custom-printed slab used was designed to maximize the chance of bioerosion and leaching of uncured photoresin, As the printed device dimensions decrease, the surface area to volume ratio increases, and thus a higher probability of bioerosion depending on the composition of the polymer resin [95].

#### 2.2.6. Microcapsule Fabrication

Microcapsules were cross-linked by ionotropic gelation, according to previously established methodology [96]. HepG2 cell suspensions in DMEM were mixed with a sterilized 3.5% (*w*/*v*) solution of alginate in DMEM to yield a final cell concentration of *N*_0_ = 1.9 × 10^6^ cells/mL of alginate. The autoclaved 3.5% (*w*/*v*) hydrogel solution was jetted into a 1.5% (*w*/*v*) CaCl_2_ bath for a cross-linking time period of 20 min. The air and liquid flowrates were adjusted to 2.5 L/min and 0.675 mL/min, respectively. The atomizer needle assembly was a concentric 24 G/16G co-axial needle (P/N 100-10-COAXIAL, Rame-Hart, Succasunna, NJ, USA,), through which the sodium alginate and air flowed. The calcified sodium-alginate beads were then washed with 0.9% (*w*/*v*) NaCl twice. The alginate-HepG2 beads were subsequently incubated at 37 °C in cell media for either 2 h (Set 1) or 24 h (Set 2) to acclimate to the 3D hydrogel environment prior to further testing. 

For the fabrication of empty alginate macrobeads that are used for compression testing, the air flowrate was reduced to 0.5 mL/min, followed by identical cross-linking and wash protocols.

#### 2.2.7. Microcapsule Compression

In order to simulate the effect of shear upon ejection from the microneedle tip, 3 mm macrobeads were subjected to surface load tests, followed by Young’s modulus and resilience calculations using Equations (1) and (2), defined by *σ*, the compressive stress (N/m^2^), *ε* the strain, *E* the Young’s modulus (N/m^2^), $E_{R}$ the resilience (J/m^3^), and $\varepsilon_{y}$ is the yield strain.

(1) σ=E × ε

(2) $$E_{R}=\overset{\varepsilon_{y}}{\underset{0}{\int }}\sigma d\varepsilon$$

A total of 15 closely packed macrocapsules that are embedded in DMEM were subjected to confined compression cycles under a 12.7 mm flat indenter using the Mach I mechanical testing systems (Biomomentum Inc., Montreal, QC, Canada) for correlating the effect of mechanical treatment to the hypothesized loss of elasticity and rupture under the following protocol: 50% compression under a load of 10 kg at a constant strain rate of 1.0 mm/s.

Following, compression macrocapsules were inspected for macro-cracks in the tens of micron range under the microscope.

Macrocapsules were used instead of microcapsules to minimize the artefacts that are associated with sample of adhesion to the indenter. 

#### 2.2.8. Optical Measurements

Microcapsule dimensioning for free and immobilized cell visualization pre and post extrusion were conducted using an Olympus CKX53 transmission microscope/camera (Olympus XM10, Center Valley, PA, USA) equipped with the cell Sens Standard imaging software. The sample size for dimension determination and the detection of rupture post extrusion was maintained at 50 capsules. This microscope was also used to measure the printed nozzle radius (r) and for all other visual inspection purposes throughout the study, unless noted otherwise.

#### 2.2.9. Microcapsule Extrusion

1.5 mL of microcapsules pooled from thee atomization batches were mixed and suspended in culture media in preparation for extrusion. Three consecutive 0.5 mL batches were loaded and extruded according to the procedure described in Section 2.2.3. at an average rate of 12 mL/min through the sterilized device from a height of 5 cm. Samples were collected in TCPS dishes and subjected to visual examination under the microscope for rupture. 

The percentage extrusion efficiency (*Ex*) given by Equation (3) was determined by normalizing the mass of 1.5 mL of extruded HepG2 capsules (*M_Ex_*) to the mass of the same volume of non-extruded HepG2 capsules (*M_C_*) used as control.

(3) $$Ex\left(\%\right)=\frac{M_{Ex}}{M_{C}}\times 100$$

#### 2.2.10. Viability Testing

Responses to extrusion shock were determined as function extrusion delay post encapsulation while using the WST-8 cell proliferation assay (ATCC, Manassas, VA, USA), as per the manufacturer’s instructions. The samples consisting of extruded and non-extruded alginate-HepG2 beads were seeded into 96-well flat bottom plates. WST-8 reagent was added to each well and the samples were incubated in a 37 °C incubator for 5h before the absorbance (*A*) was measured at 450 nm using a Tecan Infinite 200 PRO spectrophotometer (Durham, NC, USA). Background subtraction was conducted for all measurements using empty beads as controls.

#### 2.2.11. Relative Payload Calculation

Percentage relative payload (RP) given by Equation (4) was obtained by multiplying the background-subtracted absorbance (*A’*) of the sample by the extrusion efficiency (*Ex*).

(4) RP(%)=A′ ×Ex×100

#### 2.2.12. Statistical Analysis

Statistical analysis was conducted using MATLAB v2017b (Mathworks, Natick, MA, USA). One sided and two-sided sample *t*-tests were conducted at a significance level (*α*) of 0.05.

## 3. Results

### 3.1. Device Printing, Sterilization and Assembly

**Figure 3:**
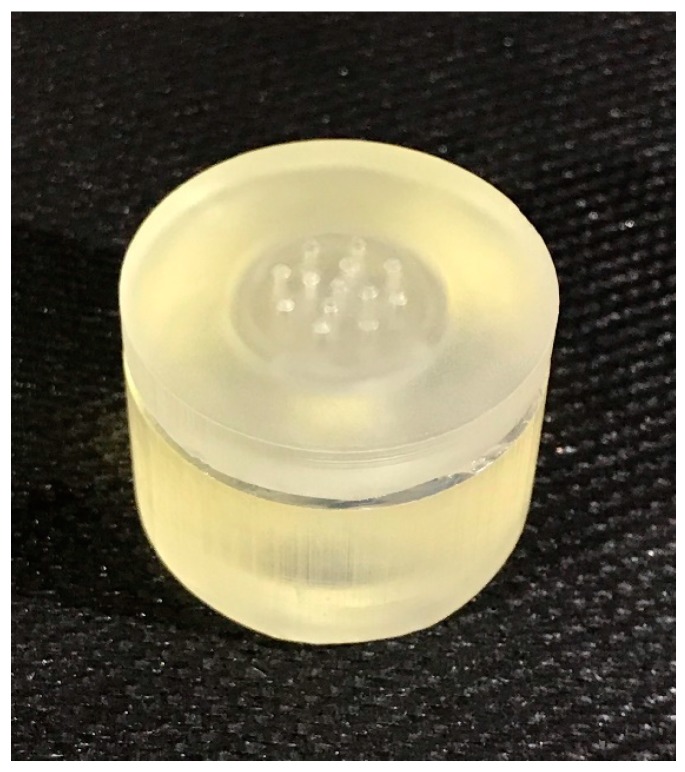
Three-dimensional (3D) printed hollow microneedle (HMN) device facing upwards.

Prior to assembly and sterilization, the array of microneedles was inspected under the microscope for dimensional analysis. The average radius (r) of the microneedle tip was measured to be 162.5 µm ± 20 µm deviating significantly from the design target of 200 µm ± 25 µm (*N* = 13, *p =* 0.01, *α* = 0.05). The assembled sterilized device is presented in Figure 3.

### 3.2. Surface Topography

**Figure 4:**
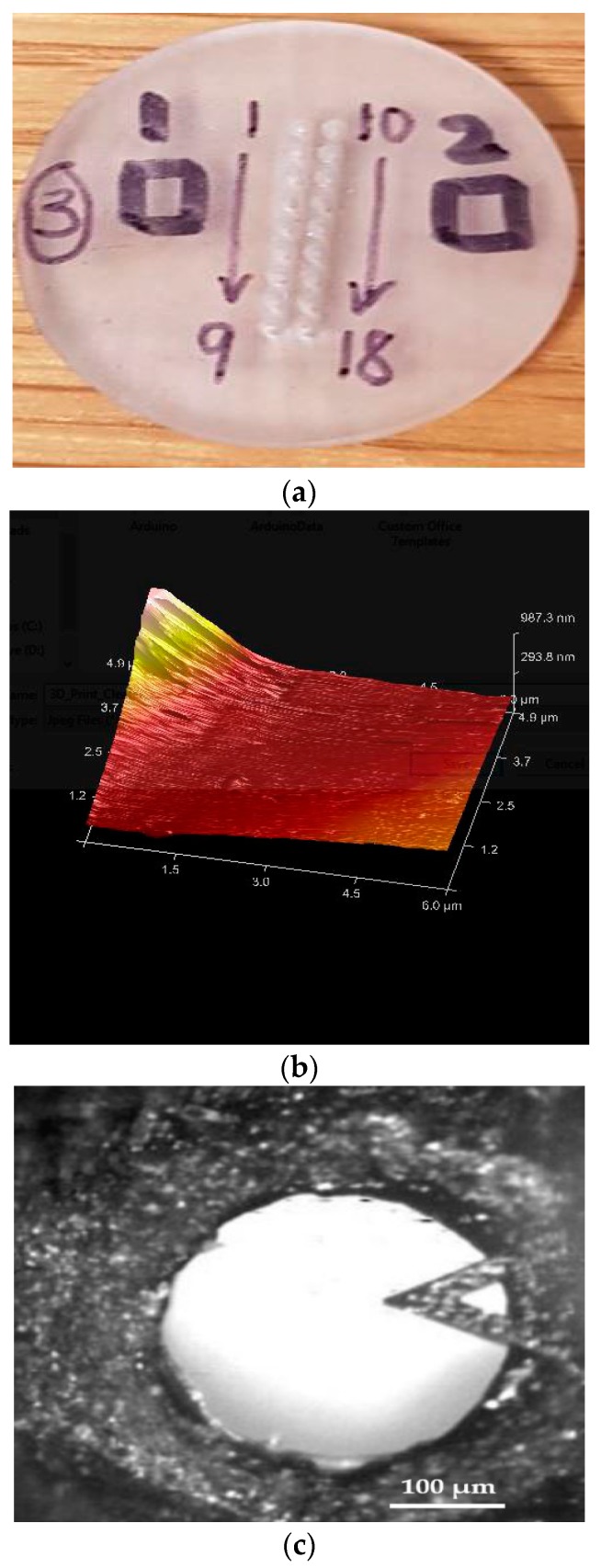

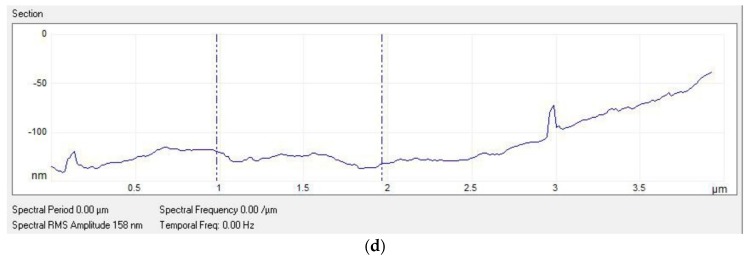
Atomic Force Microscopy (AFM) measurements: (**a**) Labeled custom-printed part designed for surface roughness measurements; (**b**) 3D structure of the regions geared towards exposure to skin surface utilized for RMS determination; (**c**) Optical Image of microneedle tip region analyzed during scan; and, (**d**) Sample AFM section analysis of microneedle tip corresponding to Figure 4c. Scale bar indicates 100 μm.

The custom-printed part designed for AFM measurements is presented in Figure 4a. The root mean square roughness (RMS) of the flat part was determined from the roughness topography (Figure 4b) generated from the 3D structure of the regions geared towards exposure to the surface of skin was 30 nm. Meanwhile, the RMS of the microneedle tip (Figure 4c) was determined to be 158 nm, as shown in the scan profile (Figure 4d).

### 3.3. Mass Flow Capability and Leakage Test

**Figure 5:**
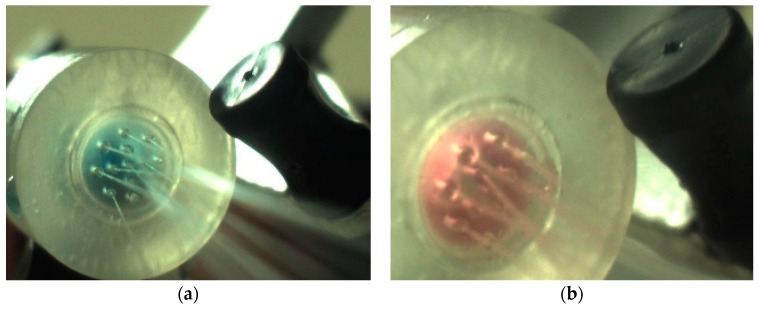
Flow testing of HMNs at 12 mL/min. (**a**) Single phase fluid blue dextran dye; and, (**b**): Two phase fluid microcapsules containing cells in Dulbecco’s modified Eagle medium (DMEM) media.

No leakage was observed with the sealed device at the lower and upper flowrates of 1.2 mL/min and 12 mL/min, respectively. At the lower flowrate, wicking was observed for both the blue dextran and microcapsules. In addition, particle aggregation resulted in nozzle occlusion and gradual total blockage for the two-phase microcapsule fluid. At the higher flowrate, jetting was observed, as captured in Figure 5, with an associated reliability of 80% over 10 runs for both fluids. Given these observations, capsule extrusion was carried at the higher flowrate of 12 mL/min for the rest of the study.

For the blue dextran, since no nozzle clogs were encountered, jetting was carried out consecutively 10 × 0.5 mL injected batches. For the microcapsules, rinsing with media was necessary after every injected batch.

### 3.4. Cytotoxicity Screening

**Figure 6:**
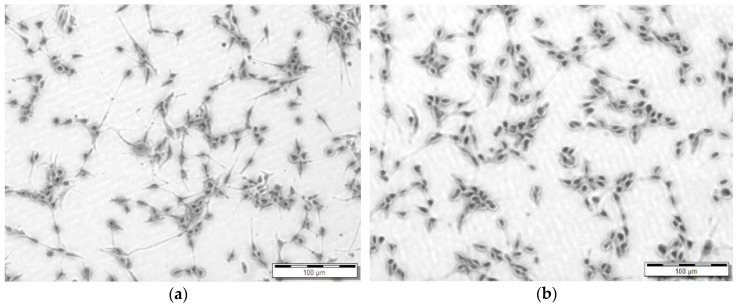
Short term Cytotoxicity Testing; (**a**) Control U87 cells after 24 h on TCPS; and, (**b**) U87 cells incubated on a 5 mm thick strip of 1 mm × 3 mm of custom 3D printed FLGPCL02^®^ photoresin at 24 h. Scale bars indicate 100 μm.

As shown in Figure 6, based on epithelial morphology and apparent confluence there is no difference in growth of U87 cells on TCPS and the 3D printed FLGPCL02^®^ photoresin.

### 3.5. Microcapsule Compression

**Figure 7:**
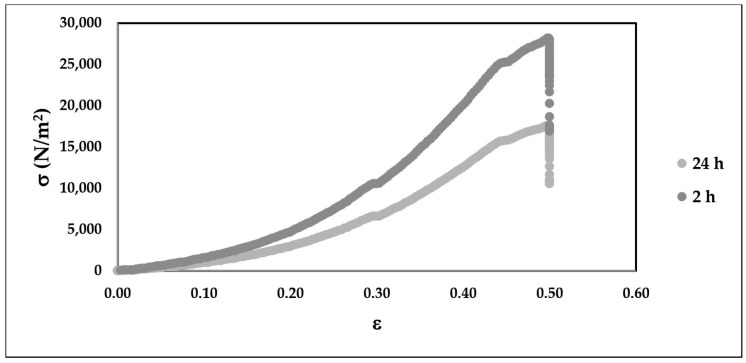
Nominal stress-strain curve for compression testing of macrocapsules 2 h and 24 h post incubation in cell media fir 50% compression at a constant strain rate of 1.0 mm/s.

The resulting nominal stress-strain curves resulting from the 50% compression post incubation at 37 °C in cell culture media based are presented in Figure 7. The yield strain was determined to be at 43% strain. The Young’s moduli for the 2 h and 24 h were 90 kPa and 56 kPa, respectively, equivalent to a resilience loss from 5470 (J/m^3^) to 3250 (J/m^3^). No macro-cracks were observed on the surface of the compressed microcapsules for either condition.

### 3.6. Microcapsule Extrusion

**Figure 8:**
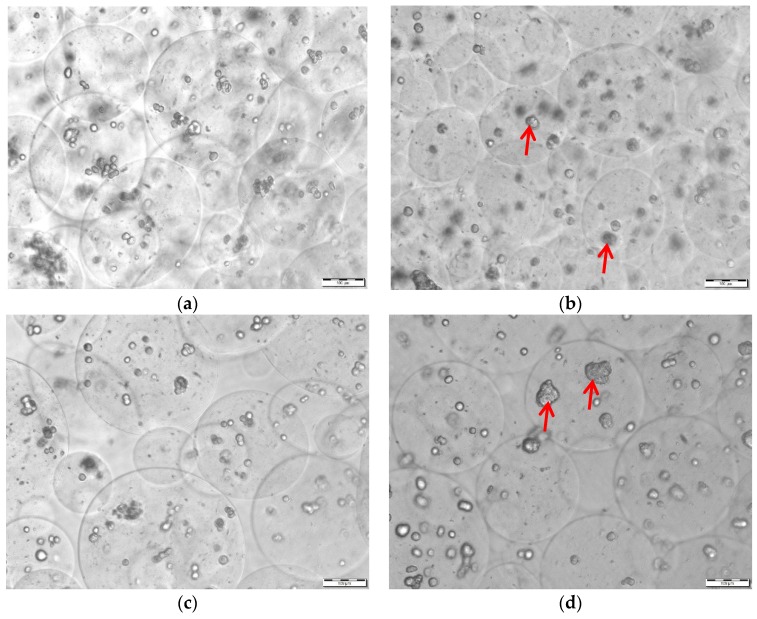
Encapsulation of HepG2 cells within alginate microcapsules. (**a**) 3.5% (*w*/*v*) alginate capsules 2 h post-fabrication (Set 1, control); (**b**) 3.5% (*w*/*v*) alginate capsules at 24 h post-fabrication (Set 2, control); (**c**) 3.5% (*w*/*v*) alginate extruded capsules 2 h post-fabrication (Set 1); and, (**d**) 3.5% (*w*/*v*) alginate capsules extruded 24 h post-fabrication (Set 2). Scale bar indicates 100µm.Red arrows represent spheroid formation.

Microcapsules that are characterized by average diameters of 225.1 µm ± 24.5 µm pre-extrusion (Figure 8a,b) were subjected to shear testing through the HMN device. Specifically, two sets of experiments comprised of 3 × 0.5 mL extrusion batches were carried out differing by the delay between cell microencapsulation and injection. 

Shown in Figure 8a–d, is a qualitative comparison of growth inside the microcapsules capsules after 2 h (Figure 8a,c) and 24 h (Figure 8b,d) of incubation. Two-hour post microencapsulation, the majority of the HepG2 cells was characterized by a rounded morphology, typical of cells that do not represent moieties for cell adhesion (Figure 8a,c). A day post-incubation, adhesion, darkening of spots, and spheroid formation became apparent (Figure 8b,d).

No rupture was detected in the extruded groups (Figure 8c,d) as compared to control (Figure 8a,b). For Set 1, where extrusion was conducted 2 h after encapsulation (Figure 8c), the extrusion efficiency was 21.2%. Shearing resulted in an uneven distribution of HepG2 capsule size (*p =* 0.00, *α* = 0.05), as characterized by an emergence of satellites (d < 100 µm). For Set 2, with a 24 h acclimation time to the 3D environment (Figure 8d) the extrusion efficiency was 54.6%. No statistically significant change in dimension (*p =* 0.18, *α* = 0.05) was detected as a result of extrusion, as illustrated in Figure 8d.

Neither swelling nor rupture was observed as a result of incubation (Figure 8a,b).

### 3.7. Viability Post Extrusion and Relative Payload

**Figure 9:**
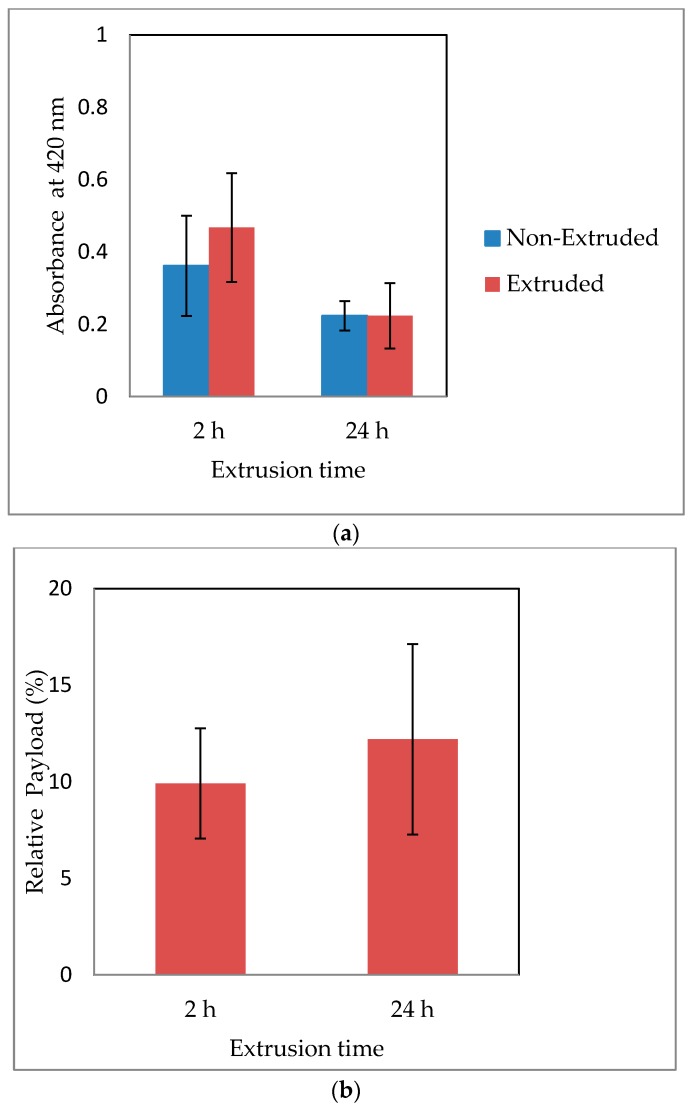
Comparative viability expressed in terms of background-subtracted absorbance units and percentage relative payload for extrusion times 2 h (Set 1) and 24 h (Set 2) post-encapsulation. (**a**) Statistical equivalence of extruded and control (Set 1, *p =* 0.14, *α* = 0.05; Set 2, *p =* 0.5, *α* = 0.05) and statistically significant decrease in viability form 2 h to 24 h (Control, *p =* 0.0298, *α* =0.05; Extruded, *p=* 0.0175, *α* = 0.05). (**b**) Statistical equivalence of percentage relative payload (2 h, RP= 9.9 ± 2.8%; 24 h, RP= 12.2 ± 4.9%, *p =* 0.2628, *α* = 0.05).

To compare the cell viability of extruded and non-extruded (control) HepG2 beads, a mitochondrial assay (WST-8) was conducted immediately post-extrusion. Results are presented in Figure 9a. Based on the results of a one-sided two sample t-test, there was no significant difference in cell viability between the extruded and control groups for Set 1 (*p =* 0.14, *α* = 0.05) and Set 2 (*p =* 0.5, *α* = 0.05). There was a significant decrease in the viability of the non-extruded control (*p =* 0.0298, *α* = 0.05) and extruded cells (*p =* 0.0175, *α* = 0.05) after a 24 h incubation period.

Viability in terms of percentage relative payload scaled to incorporate the effect of extrusion is presented in Figure 9b. The effect of extrusion delay was not found to be statistically significant on relative payload (*p =* 0.2628, *α* = 0.05).

## 4. Discussion

There is an 18.75% difference between the specified nozzle (tip) radius (r_specified_ = d/2 =200 μm) and the dimension of the printed part (r_printed_ = 162.5 μm) measured by optical microscopy. A well-documented issue of 3D printing is the resulting dimensional accuracy of a printed part. In particular, for printing methods involving layer-by-layer material solidification, such as SLA 3D printing, material shrinkage is induced by the liquid to solid phase change during the building and post-curing procedures [97]. Previously, Finite Element Analysis studies and empirical models have been developed to predict resulting printed shapes and to optimize process parameters, respectively [98], but fell short in accurate prediction due to the high complexity of the model. Although it was determined that UV-light curing provides more uniform shrinkage than thermal post-curing [99], and several studies involving DLP and SLA light-based additive manufacturing of photo-resin material observe such post-print shrinking [100,101,102], modeling, and prediction studies focusing on printed resin shrinkage have only been investigated to a certain extent [98,102]. Among such studies, Huang, et al. have developed a method to model shape volumetric shrinkage for accuracy control in additive manufacturing by plotting shrinkage under polar coordinate representation, and validated the results with SLA-derived, 3D printed SI500 photoresin by EnvisionTEC, Inc. (Dearborn, MI, USA). [98]. Alternatively, experimental volumetric shrinkage can be calculated by comparing resin density before and after it is cured. For example, Huang, et al. developed a photosensitive resin for SLA 3D printing, and measured a shrinking factor of 2.00% post-curing [103]. However, the value is highly variable among SLA printable photosensitive resins, as scan pitch and laser power of the specific 3D printer affects the shrinkage [102]. In the present study, the combined contribution of curing and alcohol washes led to a shrunk nozzle diameter of 325 µm, closer to the maximum 300 μm upper range as proposed in literature and documented to ease patient pain [63,64,65]. Future process optimization efforts will include reducing the specified nozzle diameter (d) and varying the aspect ratio of the frustum in order to achieve a value that is closer to the physical specifications for pain management stated above. Specimens will be subjected to the Izod pendulum impact test to assess the risk of using higher impact ratios [104].

Changing the aspect ratio will, in turn, enable flowrate optimization for therapeutic administration. In this study, the operating flowrate of 12 mL/min associated with reliable jetting of the bolus microcapsule suspension exceeded 40 times the therapeutic value of 0.3 mL/min [63]. Microcapsule administration at lower flowrates led to nozzle blockage induced by aggregation. Colloidal stability can be inferred by surface coating of microcapsules with a polyelectrolyte (modifying the surface charge) and changing the ionic strength of the media [105]. Since media composition is restricted by physiological requirements, physical adsorption of the alginate microcapsules by chitosan or poly-l-lysine routinely used in regenerative medicine is the only tunable variable [106]. A consequence of increased distance between capsules due to electrostatic repulsion may have a lower particle density, resulting in decreased payload. This drawback could be overcome by fabricating smaller alginate structures while using well established methodologies [107]. Another advantage of mitigating aggregation is the elimination of air use for pushing the fluid through the biodevice reservoir, which was proposed as a loading mechanism (Section 2.2.3) to prevent nozzle blockage. Following multifactorial optimization of part design, colloidal stability, and microcapsule miniaturization, with injection using a syringe pump into pig skin, the standard ex vivo model for wound healing will be investigated [108].

The exact composition of the Clear FLGPCL02^®^ methacrylate-based photoresin is proprietary. Distinguishable by clarity, the following is a non-exhaustive list of medical grade resins: dimethacrylate (DMA) [109], polymethylmethacrylate (PMMA), [110] and Methyl methacrylate/acrylonitrile/butadiene styrene (MABS) [111]. Specifically, linked to SLA are methacrylate-based proprietary photoresins [56,57,112] and acrylic-based photoresins [43,54,55]. While methacrylate-based monomers used in bone cements [113], dental fillings are considered to be biocompatible [114], their long-term implantation has been associated with irritancy and cytotoxicity. Cytotoxicity of meth (acrylates) stems from two distinct reactions [115,116]. The first one is the Michael addition, the nucleophilic addition of protein across the double bond of the acrylate function. The second one is the hydrolysis of the ester linkages, of uncured monomer or cured polymer catalyzed by carboxylesterase into carboxylic (acid)s and small molecule alcohols as principal degradation products. While, monomer degradation leads to a local decrease in pH polymer degradation, which leads to inflammation. Apart from bioerosion-related material cytotoxicity, heating of methacrylate-based thermoplastics such as laser cutting processes has been linked to ultrafine particle (UFP) generation [117]. UFP cytotoxicity has been extensively studied for the most commonly used materials in desktop 3D printers Polylactic Acid (PLA) and Acrylonitrile Butadiene Styrene (ABS). UFPs are considered a serious health concern to human health because they deposit in the pulmonary and alveolar regions of the lung [47,118]. Macdonald, et al. used the zebrafish Field Effect Transistor test to investigate the biocompatibility of photopolymers that are used in 3D printers. Leaching of uncured monomers from 3D printed parts was assessed by washing the parts within a large volume of solvents, namely ethanol (70% (*v*/*v*) and 99% (*v*/*v*)) and IPA (99% (*v*/*v*)) under agitation, with 99% ethanol yielding the highest biocompatibility results [47]. Nevertheless, it was found that the photopolymers were highly toxic due to the presence of UFP, nano-sized particles less than 100 nm in diameter, known to be released from ABS materials. PMMA biocompatibility has been proven by results of a recent 72 h cytotoxicity assessment of samples, as determined through growth monitoring, adherence, and morphology of L-929 cells in which a similar ethanol-based protocol was used for sterilization [119]. However, the above-mentioned samples were not photopolymerized, unlike the custom-printed SLA-fabricated piece in the current study. Sources for the hypothetical generation of UFPs in the current biofabrication process would be the post-curing stages and sterilization where the UFPs could be concentrated on the surface. Multiple DI rinses post-solvent sterilization were implemented to reduce the chance of UFP particle seeding. Due to the presence of the immuno-isolation membrane, and the documented molecular weight cutoff of the cross-linked alginate (MWCO) of 68 kDa, equivalent to a Stokes’ radius of 3 nm [120], in the possible event that UFPs were present on the surface of the biodevice, the viability results would not be affected. However, the risk of particulate introduction upon insertion into the patients’ skin needs to be investigated in future studies.

While the preliminary qualitative results of the short term screening (24 h) cytotoxicity presented in Figure 6, suggested an equivalency between U87 cell line proliferation on TCPS and the custom 3D printed Clear FLGPCL02^®^ photoresin, future studies will encompass an IC_50_ determination (half maximal inhibitory concentration of a substance) [94,121] using the WST-8 assay. Specifically, the following samples that were collected through sequential stages of the study incubated in DMEM will be diluted to multiple concentrations in order to construct the dose-dependent viability curve: (a) post-cured pre-assembled prototype; (b) assembled HMNs post-sterilization; and, (c) post-extruded microcapsules. PMMA surfaces are characterized by a critical surface tension of 37.5 dyn/cm [122] positioned at the lower level threshold for biofouling [123]. Surface roughness measurements that are related to bacterial adhesion research in PMMA-based implants range from 40 nm [124] to 200 nm [125] with a positive correlation between roughness and adhesion. Meanwhile, the newer bioactive bone cements are designed to be porous [126,127]. There is an overlap between the measured RMS range for the custom-printed methacrylate-based slabs (30–158 nm) and the reported biocompatible range (40–200 nm). The HMN parts in the present study were not designed to be porous. Further AFM experimentation is needed to distinguish surface roughness from random pore morphology. In addition, a Zisman plot will be constructed to determine the critical surface tension of the proprietary photoresin.

Entrapment of mammalian cells in alginate-based structures reported using various metrics depend on biofabrication methods, namely electrospraying, microfludic fabrication, in-situ polymerization, and atomization [82,128,129,130]. Use of sodium citrate for capsule core liquefaction by incubating the beads in 0.055 M sodium citrate solution (a Ca^2+^ chelator) is a standard method for cell recovery [131,132,133,134]. Because of the smaller extrusion volumes from the biodevice, the hydrogel membrane was not subjected to chelation and the viability measurements were based solely on the WST-8 assay dye diffusion through the alginate membrane. Since the Stokes’ radius of the dye is an order of magnitude smaller than the reported 70 kDa MWCO of the membrane [120], diffusion should not theoretically be rate-limiting. Shown in Figure 8a–d is the random positioning of the entrapped HepG2 cells. Due to the stagnant dye layer at the boundary of the membrane, the reaction for the cells in the middle of the capsule may have been diffusion-limited. Incubation times that are higher than 2 h recommended by the manufacturer overcame this limitation, however the accuracy of the assay was compromised by the cross-reactivity of the media. This source of error in viability determination is reflected in the norm of the standard deviations of background-subtracted absorbances shown in Figure 9a, amounting to as high as a third of the average values. There was no significant viability loss as a result of extrusion for day 1 (Set 1) and day 2 (Set 2). However, there was significant loss of viability post 24 h incubation independent of the delay between microencapsulation and extrusion. Spheroid formation that was detected after a day (Figure 8b,d) and diffusion limited transport of oxygen and nutrients due to a stagnant microenvironment may have been a root cause for this decline in viability. These hypotheses will be tested in future long-term studies, using alternate methods of viability detection, namely the live/dead fluorescence-based assays [131,134,135,136]. Furthermore, the WST-8 assay will be revisited after cell recovery by chelation of the cross-linked membrane. Once the cells are free, they may be subjected to proliferation and differentiation protocols to assess the effect of shear through the biodevice. With less noise in viability measurements, initial cell number optimization for delaying the formation of spheroids, as well as the suitability of sterilization while using the standard methods such ethylene oxide and γ-radiation will also be assessed.

Bulk and surface erosion of cross-linked alginate structures occurs in physiological media as a result of chelation and subsequent swelling [137,138]. The major constituent of FBS is bovine serum albumin [139], with a MW of 68 kDa, known to be a cutoff for cross-linked alginate membranes [120]. At constant temperature, equilibrium swelling in media is a balance between osmotic, electrostatic, and elastic forces that are holding the cross-links together [140,141]. No significant swelling was detected as a result of incubation as presented in Figure 8a,b, suggesting that either the concentration of albumin was not high enough to cause an osmotic effect after 24 h, or, the rate of chelation was insignificant for the 3.5% (*w*/*v*) alginate membrane.

It could be hypothesized that the bioerosion-driven measured a loss of resilience illustrated in Figure 7 after 24 h of incubation in media was the driving force behind the higher extrusion yield (*Ex =* 54.6%). For stiffer (*E* = 90 kPa) microcapsules that were incubated for 2 h in media (Set 1), the HMN array acted as a strict and random filter, as illustrated by the preponderance of satellites in Figure 8c and the extrusion yield of 21.2%. Meanwhile, for more flexible microcapsules (Set 2, *E* = 54 kPa) that are jetted through the HMNs change in size distribution, as previously stated, was not statistically significant (*p =* 0.18, *α* = 0.05). Elasticity of alginate microcapsules is a function of alginate composition (i.e., M/G ratio, presence of collagen), cross-linking time, matrix porosity and strain rate [134,142,143]. Furthermore, the Young’s modulus is inversely correlated to the size of the microspherical constructs assuming that adhesive contact exists between individual particles [144,145]. To that effect, the relative elasticity norms between macrocapsules and microcapsules are translatable, suggesting that it was easier to extrude softer capsules. Across the above-mentioned multifactorial studies the average Young’s modulus for 3 mm alginate beads ranged between 25–35 kPa as compared to 90 kPa obtained in the current study. Sources of discrepancy could be the higher alginate concentration of 3.5% (*w*/*v*), a higher cross-linking time of 20 min, a difference in strain rates and load cell ratings. Future parallel macro and micro indentation studies in physiological media should be conducted in DMEM in order to assess the validity of this hypothesis.

No direct comparisons can be drawn between the present study and the recent delivery of epidermal cells through silicon HMNs [88,89] due to the lack of an immuno-isolation membrane and the clinical nature of the latter research.

## 5. Conclusions

In this bench scale study a custom 3D printed HMN biodevice comprised of a reservoir chamber and an array of conical microneedle (13 conical frusta, d = 400 μm, D = 1000 μm, H = 600 μm) was fabricated using the sustainable low cost method of SLA. The Null hypothesis was accepted since the viability of extruded encapsulated HepG2 cells through the solvent-sterilized device was statistically equivalent to that of non-extruded capsules at the 95% confidence interval. With a RMS value of 158 nm being determined by AFM, the nozzle tip roughness was comparable to PMMA-based dental implants that are associated with cell adhesion. Preliminary qualitative screening cytotoxicity tests that are based on U87 cell adhesion indicated equivalency between the photoresin and TCPS in terms of biocompatibility, the validity of which should be confirmed using dose-dependent quantitative IC_50_ studies.

This microneedle platform may be customized for delivery of a broad variety of tunable scaffold properties and it should not be limited to alginate. Despite a shrinkage rate of 18.75%, the dimension of the nozzle tip measured to be 325 μm exceeded the geometric specification of <300 μm associated with patient pain minimization [63,64,65]. Using SLA and the proprietary methacrylate-based Formlabs Clear photoresin^®^, the rate limiting factor will be the improvement of printing resolution, which in turn governs flow optimization and painless administration. In the interim, the possibility of extrusion of cells protected by an immune-isolation membrane through other existing HMNs, expandable into applications of wound healing therapies pivoting on hydrogel cell interactions will be explored.

## 6. Patent

A provisional US patent (Appl. No.: 62/696410) has been filed as a result of this work.

## Supplementary Materials

The following are available online at http://www.mdpi.com/2306-5354/5/3/59/s1, Table S1: Diseases and compounds delivered using MN transdermal delivery, Table S2: MN Manufacturing methods for resorbable (RMN) and hollow (HMN) microneedles, Table S3: 3D printed Methods for MN fabrication, Table S4: MN geometries.

## Abbreviations

**Acronym**	**Definition**
ABS	Acrylonitrile Butadiene Styrene
AFM	Atomic Force Microscopy
AM	Additive manufacturing
CAD	Computer-aided design
CLIP	Continuous liquid interface production
DAB	Droplet-borne air blowing
DC-hMN-iSystem	Digitally controlled injection system
DHE	Dihydroergotamine mesylate
DMA	Dimethacrylate
DLP	Digital Light Processing
DMEM	Dulbecco’s Modified Eagle Medium
EHP	Electrohydrodynamic processing
FBS	Fetal Bovine Serum
FDM	Fused Deposition Modeling
M/G	Mannuronic acid to Guluronic acid ratio
GEN	Gentamicin
GOx	Glucose Oxidase
HA	Hyaluronic Acid
HMN	Hollow microneedle
IC_50_	Half maximal inhibitory concentration
MABS	Methyl methacrylate/acrylonitrile/butadiene styrene
MN	Microneedle
MPP	Multiphoton Polymerization
MWCO	Molecular Weight Cut Off
NP	Nanoparticles
PDMS	Polydimethylsiloxane
PEG	Poly (ethylene glycol)
PEGDA	Poly (ethylene glycol) diacrylate
PFF	Poly (propylene fumarate)
PLA	Polylactic Acid
PLGA	Poly-lactic-glycolic acid
PMMA	Polymethylmethacrylate
PVP	Polyvinylpyrrolidone
PVs	Polymeric Vesicles
RMN	Resorbable microneedle
RMS	Root mean square roughness
S/V	Surface area to volume ratio
SLA	Stereolithography
SLS	Selective Laser Sintering
TCPS	Tissue culture polystyrene
UFP	Ultrafine particle
