# Supplementary material for: Three-Dimensional (3D) Printed Microneedles for Microencapsulated Cell Extrusion
Source: Bioengineering (Basel). 2018 Jul 31;5(3):59. doi: 10.3390/bioengineering5030059 (PMC6164407; doi:10.3390/bioengineering5030059)
Supplement: Supplementary file 1 [file bioengineering-05-00059-s001.pdf]

Article

Three-Dimensional (3D) Printed Microneedles for Microencapsulated Cell Extrusion

Chantell Farias [1], Roman Lyman [1], Cecilia Hemingway [1], Huong Chau [1], Anne Mahacek [2], Evangelia Bouzos [1] and Maryam Mobed-Miremadi [1,*]

[1] Department of Bioengineering, Santa Clara University, Santa Clara, CA 95053-0583, USA; cfarias@scu.edu (C.F.); rlyman@scu.edu (R.L.); chemingway@scu.edu (C.H.); hchau@scu.edu (H.C.); ebouzos@scu.edu (E.B.)

[2] SCU Maker Lab, Santa Clara University, Santa Clara, CA 95053-0583, USA; amahacek@scu.edu (A.M.)

* Correspondence: mmobedmiremadi@scu.edu; Tel.: +408-554-2731

Table S1. Diseases and compounds delivered using MN transdermal delivery.

Disease	Compound(s) delivered	Method	MN dimensions	Study
Melanoma	pH-sensitive dextran nanoparticles (NPs) loaded with anti-PD1 antibody, GOx	HA-based transdermal MN array integrated with NPs	15 × 15 array of conical MNs of 600 µm height, 300 µm base diameter, sharp tip tapering to 5 µm radius of curvature in 600 µm tip-to-tip spacing in 81 mm^2 patch	Wang, et al. [2]
Melanoma	Tumor lysate, melanin	Infrared-irradiated, tip-methacrylated, HA-based MN array loaded with tumor lysate and melanin	15 × 15 array of conical MNs of 800 µm height, 300 µm base thickness, sharp tip tapering to 5 µm in 81 mm^2 patch	Ye, Y., et al. [3]
Type I Diabetes	Glucose-responsive polymeric vesicles (PVs) loaded with insulin, glucose oxidase (GOx)	Hyaluronic acid (HA) based transdermal MN array integrated with PVs	20 × 20 array of conical MNs of 600 µm height, 600 µm tip- to-tip spacing in 100 mm^2 patch	Hu, et al. [4]
Type II Diabetes	Exendin-4 (Ex-4) hormone for glycaemic control	Dissolving MNs encapsulated with Ex-4	3 × 3 array of conical MNs of 450 µm height, 35 µm tip diameter	Lahiji, et al. [5]
Hepatitis B	Cationic liposomes encapsulated with hepatitis B DNA vaccine VR-E2E and adjuvant CpG oligonucleotides	Polyvinylpyrrolidone (PVP-K17) dissolving MN array loaded with cationic liposomes	6 × 6 array of tetrahedral MNs of 650 µm height, 250 µm base length, 450 µm tip-to-tip spacing in 36 mm^2 patch	Qiu, et al. [6]
Tetanus	Unadjuvanted tetanus toxoid for vaccination	MN array formed by casting vaccine solution (PVA, sucrose, CMC in	10 × 10 array of conical MNs of	Esser, et al. [7]

		dibasic potassium phosphate buffer pH 7.4) containing tetanus toxoid monobulk	650 μm height, 250 μm base diameter	
Human Papilloma Virus warts	Bleomycin	Polylactic Acid-based, Bleomycin tip-coated MN array	10 × 10 array of pyramidal MNs of 650 μm height, 250 μm base diameter, in 49 mm^2 patch	Ryu, et al. [8]
Human Papilloma Virus positive cancers	Cationic liposomal HPV $E7_{43–63}$ synthetic long peptide vaccine for host anti-tumor immune response	Silica capillary based single hollow MN and digitally controlled injection system (DC-hMN-iSystem)	Fused silica capillary of length 400 μm, 50 μm inner lumen diameter, 149 μm lumen diameter at tip, 66 μm bevel length	Maaden, et al. [9]
Breast cancer	Tamoxifen, Gemcitabine chemotherapeutic agents	Zein-based MN array coated with Tamoxifen or Gemcitabine, poke and patch administration	6 × 6 array of conical MNs of height and width 965 ± 23 and 363 ± 15 μm, respectively	Bhatnagar, et al. [10]
HPV-induced Cervical cancer	NPs containing RALA-E6/E7 proteins for DNA vaccination	Polymeric polyvinylpyrrolidone (PVP)-based MN loaded with RALA-E6/E7 NPs	19 × 19 array of conical MNs of 600 μm height, 300 μm base width, and 300 μm interspacing	Ali, et al. [11]
Neonatal sepsis infection	Gentamicin (GEN)	PVA, PVP. PEG-based dissolving MN array containing GEN	19 × 19 array of pyramidal MN of 500 μm height and 0.45 cm^2 area	González-Vázquez, et al. [12]
Mycobacterium Tuberculosis	Bacillus Calmette–Guerin polysaccharide nucleic acid (BCG-PSN) powder	BCG-PSN powder-laden sodium HA-based MN patch	6 × 9 array of conical MN of 200 μm height and 100 μm base diameter	Yan, et al. [13]
Obesity	Rosiglitazone (Rosi) browning agent, GOx, catalase encapsulated in NPs	Rosi NP-embedded and HA-based MN patch	11 × 11 array of conical MN of 800 μm height, 300 μm base diameter, 600 μm tip-to-tip spacing in 49 mm^2 area	Zhang, et al. [14]
Osteoporosis	Alendronate	Alendronate tip-coated HA-based dissolving MN array	190 array of MN of 800 μm height, 40 μm tip diameter,	Katsumi H., et al. [15]

Acute migraine	Dihydroergotamine mesylate (DHE)	DHE-loaded, PVP-based dissolving MN array	160 μm base diameter<br><br>10 × 10 array of conical MN of 1 cm^2 area	Tas, et al. [16]

Table S2. MN Manufacturing methods for resorbable (RMN) and hollow (HMN) microneedles.

Process	Method	Example Study	Fabricated dimensions
RMN Fabrication			
Micromolding	Addition drying/photocrosslinking of polymer mixture into PDMS mold fabricated by lithography/etching/thermal drawing techniques	González-Vázquez, et al. [12]	19 × 19 array of pyramidal MN of 500 μm height and 0.45 cm^2 area
Direct photolithography	Polymer casting into PDMS mold, UV exposure, and cross-linking with photocatalyzer	Dardano, et al. [30]	Array of cylindrical MNs of 150 μm height and conical, lancet-shaped MNs of 150 to 2240 μm height
Drawing lithography	Polymer is melted, dispensed on a plate, moving up and down to elongate drawn pillars	Choi, et al. [31]	7 × 7 array of HA-based conical MN of 350 μm height, 380 μm base diameter, and 30 tip diameter μm
Solvent casting	Drug of interest cast onto PDMS laser-engrafted mold and dried under vacuum, then polymer solution layer cast onto mold, and base plate pasted onto polymer solution after freeze-drying and extraction from mold	Wang, et al. [32]	5 × 5 array of conical MN with height of 600 μm and base diameter 300 μm
Electro-drawing	Deformation of PLGA solution sessile drops by application of electrohydrodynamic force, with drug preloaded polymer solution kept at mild temperature	Ruggiero, et al. [35]	Arrays of 10 × 10 cylindrical pillars, of tip-to-tip distance of 1.2 mm, base diameters ranging from 300 μm to 600 μm, and height of 500 μm
Continuous liquid interface production (CLIP)	Photoreactive resin photopolymerized on rising platform by Ultra-violet (UV) beam that passes through transparent window at bottom of resin to selectively target and solidify into MNs	Johnson, et al. [36]	Array of square pyramidal MN of 1mm height, 300 μm width
Droplet-borne air blowing (DAB)	Polymer drug mixture dispensed as droplet, contacted and drawn for biconcave	Huh, et al. [37]	3 × 3 array of MN, height of 280 ± 10 μm (epidermal

	shape, then air-dried and separated		growth factor MN), 30 ± 5 μm (ascorbic acid MN).
Dipping	Polymer-coated pillar tips coated in drug of interest dipped in drug-unmixed solution, then lifted, air-dried, and separated to form MN tips	Kim, et al. [38]	5 × 5 array of conical MN, height of 100 μm and 170 μm diameter
3D printing	Formation of 3-dimensional structure from 2-dimensional design by construction methods such as extrusion, sintering, binding	Luzuriaga, et al. [39]	5 × 5 array of MN of height range 200–2,500 μm, width 400–600 μm, thicknesses 400–600 μm, and tip diameters from 170–220 μm
HMN Fabrication			
Micromolding	Mold created from a master structure, then creation of replicas through sputtering, deposition, and dissolving of material to form a replica of the structure	Norman, et al. [29]	Single tapered cylindrical MN of 1.1 mm tall, 225 μm in radius at the base, and 20 μm in radius at the tip
Mold-based Etching	Gradual deposition and etching of films onto silicon wafers to create pattern indents for molding	Kim, et al. [33]	13 × 13 array of 40 μm in diameter consisting of hollow microneedles of 250 μm in height, 140 μm in base width, 1 mm in pitch, and 40 μm in borehole
Lithography	Silicon wafer coated with photoresist, UV-exposed, then covered with mask to form design	Ceyssens, et al. [34]	5 × 5 array of triangle cylindrical MN; height of 1mm and base of 175 μm
Laser micromachining	MN laser-cut from stainless steel sheets	Vinayakumar, et al. [40]	13 × 13 array of MN; 1 mm in pitch, 160 μm in base width, approximately 250 μm in height
Solvent casting	Electro-deposition onto PDMS laser-engrafted mold and dried under vacuum, then extraction from mold	Mansoor, et al. [41]	Array of 500 μm tall MNs with a tip lumen diameter of 40 μm and tip wall thickness of 15 μm

Table S3. 3D printed Methods for MN fabrication.

Process	Method	Material	Study
Vat Photopolymerization	Continuous liquid interface production (CLIP)	Trimethylolpropane Triacrylate	Johnson, et al. [36]
Extrusion	Fused Deposition Modeling (FDM™)	Polylactic Acid (PLA)	Luzuriaga, et al. [39]
Vat Polymerization	Multiphoton polymerization (MPP)	Polymer-ceramic hybrid (containing urethane- and thioether (meth)-acrylate alkoxysilanes) by Ormocer, Inc.	Ovsianikov, et al. [42]
Vat Polymerization	Stereolithography (SLA)	Bovine serum albumin-containing Poly (ethylene glycol) diacrylate (PEGDA) hydrogel	Kang et al. [50]
Vat Polymerization	SLA	Poly(propylene fumarate)/diethyl fumarate biodegradable photopolymer loaded with dacarbazine	Lu, et al. [51]
Powder Bed Fusion	Selective Laser Sintering (SLS)	Biocompatible stainless steel alloy 316L	Gieseke, et al. [52]
Vat Polymerization	Digital Light Processing (DLP)	eShell 200 photosensitive acrylate-based biocompatible polymer resin by Envisiontec, Inc.	Miller, et al. [54]
Vat Polymerization	SLA	R11 acrylate based photopolymer by Envisiontec, Inc.	Lacan, et al. [57]
Vat Polymerization	SLA	Class I photopolymer resin by Formlabs®	Pere, et al. [58]
Vat Polymerization	SLA	FLGPCL02 photopolymer resin by Formlabs®	Kundu, et al. [112]
Vat Polymerization	SLA	VisiJet FTX Clear Photocurable resin (50% triethylene glycoldiacrylate, 20% isobornyl methacrylate, 2% phenylbis(2,4,6-trimethyl benzoyl)- phosphine oxide) by 3D Systems, Inc.	Liu, et al. [55]
Vat Polymerization	DLP	eShell 200 photosensitive acrylate-based biocompatible polymer resin by Envisiontec, Inc.	Boehm, et al. [43]
Vat Polymerization	DLP	3DM Cast photopolymer resin by Kudo3D, Inc.	Lim, et al. [44]
Vat Polymerization	DLP	Photosensitive photoresist	Faraji Rad, et al. [45]

Table S4. MN geometries.

Geometry	MN dimensions	Array dimensions	Study
Conical	600 μm height, 300 μm base diameter, sharp tip tapering to 5 μm radius of curvature in 600 μm tip-to-tip spacing	15 × 15 array in 81 mm^2 patch	Wang, et al. [2]
Conical	600 μm height, 600 μm tip- to-tip spacing	20 × 20 array in 100 in 100 mm^2 patch	Hu, et al. [4]
Pyramidal	650 μm height, 250 μm base diameter	10 × 10 array in 49 mm^2 patch	Ryu, et al. [8]
Pyramidal	500 μm height and 0.45 cm^2 area	19 × 19 array of pyramidal MN of	González-Vázquez, et al. [12]
Conical	800 μm height, 300 μm base diameter, 600 μm tip-to-tip spacing	11 × 11 array in 49 mm^2 area	Zhang, et al. [14]
Tip-beveled triangular cylinder	1 mm height, and triangular cross-section and base of 175 μm, 54.74° tip bevel	5 × 5 array	Ceyssens, et al. [34]
Cylindrical body, pointed head	Cylindrical base of 700 μm in length, conical tip of 300 μm in length; 20 μm diameter of the apex of the cone, 200 μm base diameter. 0.58 mm^2 surface area and 0.33 mm^3 volume	25 MN where individual microneedles had a 2 × 2 mm substrate with a thickness of 300 μm	Lu, et al. [51]
Triangularly-sloped	Triangular base with 1.2 mm sides, height of 1.5 mm, and vertical cylindrical channel of diameter 400 μm (hollow)	2 × 2 array with 2 mm inter-needle spacing	Miller, et al. [54]
Cylindrical	Inner diameter 160 μm, outer diameter 220 μm, height 900 μm (hollow)	4 × 4 array in 4 mm^2 patch	Liu, et al. [55]
Cylindrical body, pointed head	First design: 700 μm total height, 150 μm tip height, 150 μm flange height;<br>Second design: 700 μm total height, 350 μm tip height, 150 μm flange height.	First design: 16 microneedle array (2.17 mm×2.17 mm)<br>Second design: 16 microneedle array (2.17 mm×2.17 mm)	Faraji Rad, et al. [45]
Cylindrical body, pointed head	Body: 400 μm in height, 300 μm in diameter<br>Tip: 100 μm height	6 × 6 array with 1 mm inter-needle spacing	Nagamine, et al. [56]
Tip-beveled cylinder	2 mm height, 60 μm inner diameter and 100 m outer diameter, laser-cut 30° and 60° bevel angles	Single needle for blood extraction	Lee, et al. [59]
Tip-beveled cylinder	980 μm height, 300 μm wide shaft; 590 μm distance between lumen opening and baseplate; 150 μm diameter of the lumen opening	6 × 6 array with 900 μm spacing between MN	Wang, et al. [60]

Pyramidal	500 μm height, 300 μm base diameter	8 × 8 array with 500 μm spacing between MN	Lau, et al. [61]
Tetrahedron-shaped	500 μm in height (produced by Elegaphy, Inc.)	3 × 9 array	Nguyen, et al. [62]

© 2018 by the authors. Submitted for possible open access publication under the terms and conditions of the Creative Commons Attribution (CC BY) license (http://creativecommons.org/licenses/by/4.0/).
